# Geographical and racial and/or ethnic disparities in pediatric ARDS mortality in the USA, 2016–2022: a triennial national database retrospective cohort analysis

**DOI:** 10.1016/j.lana.2025.101355

**Published:** 2025-12-30

**Authors:** Garrett Keim, Paula Magee, Cody Gathers, Anireddy R. Reddy, Charlotte Z. Woods-Hill, Nadir Yehya

**Affiliations:** a Department of Anesthesia and Critical Care Medicine, University of Pennsylvania Perelman School of Medicine, Philadelphia, PA, USA; b Division of Pediatric Critical Care Medicine, Children's Hospital of Philadelphia, Philadelphia, PA, USA; c Leonard Davis Institute for Health Policy Economics, University of Pennsylvania, Philadelphia, PA, USA; d Division of Pediatric Cardiology, Children's Hospital of Philadelphia, Philadelphia, PA, USA

**Keywords:** Pediatric acute respiratory distress syndrome, PARDS, Disparities, Pediatric critical care

## Abstract

**Background:**

Disparities in pediatric critical care outcomes are recognized, but national data describing Pediatric Acute Respiratory Distress Syndrome (PARDS) prevalence, mortality and temporal trends are limited. We described prevalence, and regional and racial/ethnic mortality disparities for algorithm-defined ARDS, a surrogate for PARDS in US children from 2016 to 2022.

**Methods:**

We performed a retrospective cohort study using the 2016, 2019, and 2022 Kids' Inpatient Database (KID). Algorithm-defined ARDS was identified with an ICD-10 approach requiring acute respiratory failure from pulmonary, sepsis, or shock etiologies requiring invasive mechanical ventilation ≥24 h. The primary outcome was in-hospital mortality. Exposures were US region and Race/Ethnicity, modeled individually and jointly. Mixed-effect logistic regression models, adjusting for income quartile, APR-DRG severity of illness, hospital type, and complex chronic conditions, estimated adjusted mortalities and risk differences.

**Findings:**

Algorithm-defined ARDS occurred in about 42,000 hospitalizations per year, with prevalence increasing from 0.68% (95% CI 0.67–0.69) in 2016 to 0.75% (0.74–0.75) in 2022. Overall mortality was 12.9% (12.5–13.3) in 2016, 12.5% (12.1–12.9) in 2019, and 13.7% (13.3–14.1) in 2022. In the joint model, relative to Northeastern White children (predicted 10.9%, 95% CI 9.72–12.1), risks were higher for Black children in the South (predicted 14.2%, ARD 3.27%, 1.74–4.79) and West (14.6%, ARD 3.69%, 1.39–6.00); Hispanic children in the West (12.6%, ARD 1.70%, 0.09–3.31), and children of Other race/ethnicity in the South (16.5%, ARD 5.57%, 3.14–7.99) and West (14.0%, ARD 3.11%, 0.96–5.25). Disparities did not meaningfully change from 2016 to 2019, while mortality increased from 2019 to 2022.

**Interpretation:**

Algorithm-defined ARDS among hospitalized US children remains common and highly fatal. Persistent regional and racial/ethnic disparities highlight systemic drivers of inequity and the need for targeted interventions.

**Funding:**

This work was supported by the 10.13039/100000050National Heart, Lung, and Blood Institute, National Institutes of Health (Award K23HL177271, PI: Keim).

**Research in context**

**Evidence before this study**

We searched PubMed/MEDLINE, and Google Scholar for studies published from Jan 1, 2000 to January 1, 2025, without language restrictions. Search terms combined subject headings and keywords related to pediatric acute respiratory distress syndrome or ARDS and prevalence, incidence, and regional or racial disparities.

Available evidence shows that PARDS is uncommon at the population level, but accounts for a meaningful proportion of PICU admissions, with an incidence of 3% overall and 6% among invasively ventilated patients in a prospectively performed study after the first pediatric definition was published. Overall mortality typically ranges from 12% to 20%, with higher risk in subgroups such as severe PARDS, sepsis, trauma, and immunocompromised subjects. We found no United States nationally representative analyses spanning the period since the first published PARDS definition.

**Added value of this study**

Using the three most recent releases of the Kids’ Inpatient Database (2016, 2019, 2022) and a validated ICD-10 algorithm to identify a surrogate for PARDS, algorithm defined-ARDS, we provide the first U.S. population-level estimates of prevalence and in-hospital mortality since original definition publication and across the pre- and post-pandemic period. We demonstrate a small but measurable rise in prevalence and a post-2019 increase in mortality. We characterize high-risk subgroups including severe sepsis, trauma, malignancy and complex chronic conditions and hospitalization characteristics for algorithm-defined ARDS including length-of-stay and inflation adjusted hospital charges. Further, we quantify disparities by U.S. region and by race and/or ethnicity using both separate and joint exposure models with adjustment for median household income, hospital type, and medical comorbidities. This work improves the generalizability of prior epidemiologic estimates by offering nationally representative prevalence and explores important aspects of disparate outcomes for children with algorithm-defined ARDS.

**Implications of all the available evidence**

Taken together with prior literature, our findings indicate that the national burden of PARDS is not diminishing and that mortality rose after 2019. Long-standing disparities by U.S. geographic region and race and/or ethnicity persisted without evidence of improvement between 2016 and 2022. Policy and practice should prioritize equitable access to pediatric critical care (including referral/transfer networks and regional capacity planning) and targeted quality-improvement in vulnerable regions. Future research should shift from documenting gaps to further delineating mechanisms behind these disparities and testing solutions to improve the care of critically ill children with PARDS.

## Introduction

Despite improving mortality,^1^ Pediatric Acute Respiratory Distress Syndrome (PARDS) remains one of the most fatal conditions cared for in pediatric intensive care units (PICUs) with a recently published North American overall mortality of 15%.^2^ Survivors are at high risk for morbidity and readmission.^3^, ^4^, ^5^ Following publication of the 2015 Pediatric Acute Lung Injury Consensus Conference (PALICC) definition, the Pediatric Acute Respiratory Distress Syndrome Incidence and Epidemiology (PARDIE) study established the first prevalence data for pediatric ARDS among patients in the PICU, occurring in 3.2% of all children in the PICU and 6.1% of patients receiving mechanical ventilation.^2^ Studies evaluating trends in prevalence and mortality of PARDS since creation of the PALICC definition, and particularly pre- and post-COVID era, do not exist.

Racial, ethnic, and socioeconomic disparities in outcomes for sepsis and pneumonia, two common inciting insults for PARDS, are well documented.^6^^,^^7^ Black children have been shown to experience higher mortality from sepsis, particularly in the Southern and Western United States (US), even after accounting for illness severity and socioeconomic factors.^6^ Similarly, racial and ethnic disparities in community acquired pneumonia (CAP) mortality are influenced by geographic region and age, with Black infants in the South and West demonstrating notably higher mortality.^7^ These persistent disparities underscore deep-seated systemic issues that adversely affect outcomes for vulnerable pediatric populations.

Despite this knowledge for related conditions, there remains a critical need for contemporary, national-level data on PARDS prevalence and mortality using a consistent and validated definition.^4^ More importantly, it is largely unknown whether geographical and racial and/or ethnic disparities in PARDS mortality have evolved over time, particularly against a backdrop of increasing national discourse on health equity.

The Healthcare Cost and Utilization Project (HCUP) Kids' Inpatient Database (KID) is the largest, all-payer, nationally representative pediatric inpatient database, capturing hospitalizations from a broad spectrum of community, academic, and freestanding children's hospitals in the US. KID is a stratified systematic random sample of pediatric discharges from participating states (sampling 10% of uncomplicated births and 80% of other pediatric cases) and provides weights to generate national estimates. Interhospital transfers are treated as distinct records that cannot be matched across hospitalizations. Its sociodemographic, payer, and hospital-level variables enable evaluation of temporal trends in patient mix, hospital characteristics, and outcomes at both national and regional levels. Identification of syndromic diagnoses such as PARDS using International Classification of Diseases, Tenth Revision (ICD-10) codes is challenging. We previously developed and validated an algorithm using ICD-10 diagnosis and procedure codes to identify a surrogate for ARDS and quantified its performance characteristics in a local pediatric cohort with clinician confirmed invasively mechanically ventilated ARDS.^4^ The inclusion of both ICD-10 diagnosis and procedure codes in KID allows us to apply this algorithm at scale to identify algorithm-defined ARDS hospitalizations and evaluate national patterns in prevalence, outcomes, and disparities.

This study has three primary objectives: (1) To describe the national prevalence of algorithm-defined ARDS and associated in-hospital mortality among US pediatric hospitalizations triennially in the 2016 to 2022 HCUP KID; (2) To detail patient and hospital characteristics associated with algorithm-defined ARDS hospitalizations over time; and (3) To assess trends in geographical and race and/or ethnicity disparities in algorithm-defined ARDS mortality. We hypothesized that geographic and racial and ethnic mortality disparities would persist between 2016 and 2022.

## Methods

### Study design and data source

We performed a retrospective cohort study of children with algorithm-defined ARDS, as determined by a previously published ICD-10 coding algorithm (Supplemental Table S1).^3^^,^^4^ During data analysis, we used ChatGPT (OpenAI; GPT-o3 and GPT-5 Thinking) to help draft and debug Stata code for mixed-effects logistic regression and data visualizations by iteratively providing descriptions of variables, model specifications, and error messages and requesting corrected code. All outputs were independently reviewed, validated against Stata documentation, and results were reproduced from the final analyst-written scripts.

### Exposure and outcome variables

The primary outcome was all-cause in-hospital mortality. Race and/or ethnicity was categorized into Non-Hispanic White (hereafter White), Black, Hispanic (KID assigns subjects as Hispanic if either race and/or ethnicity was input as “Hispanic”), and Other. The “Other” category includes Asian or Pacific Islander, Native American, and other/multiracial individuals due to small numbers in some subgroups. Race and/or ethnicity were obtained from HCUP partner-supplied fields; reporting practices varied across hospitals and may reflect self-report or hospital staff entry. Complete case analysis was used for regressions that had race and/or ethnicity as an exposure (Supplemental Figure S1). Covariates for adjustment in the multivariable models included: median household income quartile for the patient's residential ZIP code; All Patient Refined Diagnosis Related Groups (APRDRG) severity of illness; hospital type (rural, urban non-teaching, urban teaching); and the presence of one or more complex chronic conditions (CCCs), identified using the pediatric CCC classification system version 2.^8^ APRDRG severity of illness is a 4-level categorical variable that utilizes subject base APRDRG, severity illness subclass, and risk of mortality to categorize patients from minor or no loss of function to extreme loss of function or death. Total hospital charges were inflation-adjusted to 2022 US dollars.^9^

### Statistical analysis

Descriptive statistics, including frequencies and percentages for categorical variables and medians with interquartile ranges (IQRs) for continuous variables, were used to characterize algorithm-defined ARDS prevalence, mortality, patient demographics, hospital characteristics, and risk factors for each study year (2016, 2019, 2022). HCUP-provided discharge weights were utilized to account for the sampling probability of each stratum, allowing for national estimates of disease burden to be generated from the raw counts. All descriptive statistics utilized the provided weighting.

To assess geographical and racial and/or ethnic disparities in algorithm-defined ARDS mortality and their trends over time, mixed-effect logistic regression models were employed, with in-hospital mortality as the binary outcome. First, separate unadjusted and adjusted models to evaluate US Region (Northeast, South, Midwest, and West), with Northeast as the reference group, based on the lowest predicted mortality and previous findings in sepsis and CAP,^7^^,^^10^ and race and/or ethnicity (White, Black, Hispanic, Other), with White as the reference group were performed. Secondly, a joint exposure (US region and race and/or ethnicity) variable was created. Unadjusted and adjusted mixed effect regressions were performed with White children in the Northeast serving as the reference group which was chosen because it had the lowest observed algorithm-defined ARDS mortality in the dataset, allowing risk differences to be interpreted as excess risk relative to the subgroup with the lowest mortality. We fit mixed-effects logistic regression models (Stata melogit) with discharge weights and hospital identification number as a random effect in all models to account for the clustering of patients within hospitals and then converted coefficients to risks via marginal standardization using margins (reporting 95% CIs from the delta method). Absolute risk differences (ARDs) were obtained as average marginal effects for factor levels versus the prespecified reference. Complete-case regressions were utilized for primary analyses; as a sensitivity analysis, missing race/ethnicity was multiply imputed using chained equations (multinomial logit including the outcome and covariates; 40 imputations), with estimates pooled via Rubin's rules.

To evaluate changes in disparities over the study period, year-specific models were run, and the ARD and their 95% CIs were compared across the years 2016, 2019, and 2022. Trend over time was evaluated using the interaction between year and the primary exposure variable in each of the three confounder adjusted models. All analyses were conducted using Stata (StataCorp LLC, College Station, TX, USA).

### Ethical considerations

This study utilized de-identified data from the publicly available HCUP KID database. As such, it was considered non-human subjects research by the institutional review board of the Children's Hospital of Philadelphia.

### Role of the funding source

Funders had no role in study design, data collection, data analysis, interpretation, writing of the report or decision to submit.

## Results

### Prevalence and mortality of algorithm- defined ARDS

In 2016, there were 6,308,824 weighted pediatric hospitalizations, among which 42,831 cases of algorithm-defined ARDS were identified. In 2019, out of 5,897,361 hospitalizations, 42,407 algorithm-defined ARDS cases were identified, and in 2022, from 5,621,333 hospitalizations, 42,160 algorithm-defined ARDS cases were identified. The overall prevalence of algorithm-defined ARDS among all pediatric inpatient hospitalizations increased from 0.68% (95% CI 0.67–0.69) in 2016, to 0.72% (0.71–0.73) in 2019, and further to 0.75% (0.74–0.75) in 2022 (Table 1) (Supplemental Figure S2). When restricted to non-birth hospitalizations, the prevalence was higher and increased over time: 1.58% (1.56–1.60) in 2016, 1.69% (1.67–1.71) in 2019, and 1.83% (1.81–1.85) in 2022. Overall, in-hospital mortality for children with algorithm-defined ARDS was 12.9% (95% CI 12.5–13.3) in 2016, 12.5% (12.1–12.9) in 2019, and increased to 13.7% (13.3–14.1) in 2022.

**Table 1:** Triennial prevalence and mortality of pediatric algorithm- defined ARDS among inpatient hospitalizations.

Reason for hospitalization	2016% prevalence (95% CI) [N]	2019% prevalence (95% CI) [N]	2022% prevalence (95% CI) [N]	2016% mortality (95% CI) [N]	2019% mortality (95% CI) [N]	2022% mortality (95% CI) [N]
All hospitalizations	0.68% (0.67–0.69) [N = 42,831]	0.72% (0.71–0.73) [N = 42,407]	0.75% (0.74–0.75) [N = 42,160]	12.9% (12.5–13.3) [N = 5532]	12.5% (12.1–12.9) [N = 5292]	13.7% (13.3–14.1) [N = 5780]
Non-birth hospitalizations	1.58% (1.56–1.60) [39,385]	1.69% (1.67–1.71) [39,510]	1.83% (1.81–1.85) [39,297]	11.97% (11.60–12.35) [4712]	11.43% (11.07–11.79) [4512]	12.67% (12.29–13.06) [4978]
Severe sepsis	30.80% (29.97–31.65) [4970]	27.42% (26.68–28.17) [5095]	27.66% (26.92–28.42) [5167]	23.58% (22.21–25.00) [1170]	22.02% (20.73–23.38) [1122]	23.35% (22.02–24.73) [1205]
Other shock	15.75% (15.40–16.10) [9292]	14.41% (14.12–14.71) [10,329]	15.12% (14.83–15.42) [11,684]	22.14% (21.15–23.15) [2054]	20.69% (19.80–21.61) [2137]	22.14% (21.27–23.03) [2585]
Pneumonia	6.82% (6.68–6.96) [11,500]	6.63% (6.50–6.77) [11,891]	7.07% (6.94–7.21) [12,780]	8.09% (7.52–8.70) [930]	7.09% (6.57–7.64) [842]	7.75% (7.23–8.31) [990]
Bronchiolitis	3.86% (3.75–3.98) [5996]	3.87% (3.77–3.98) [7098]	3.29% (3.21–3.38) [7071]	2.82% (2.36–3.36) [169]	1.99% (1.65–2.40) [141]	2.34% (1.96–2.79) [165]
Trauma	–	5.13% (5.02–5.24) [11,463]	5.15% (5.03–5.24) [11,425]	–	15.21% (14.46–15.99) [1741]	16.5% (15.70–17.29) [1881]

Algorithm-defined ARDS developed frequently in children hospitalized for severe sepsis (prevalence 30.8% in 2016, 27.4% in 2019, and 27.7% in 2022) with high mortality in this subgroup (23.6% in 2016, 22.0% in 2019, and 23.4% in 2022) (Table 1). A KID identification of hospitalization with a traumatic injury code was present in about 5% of algorithm-defined ARDS cases, with a mortality over 15% in subjects with this code (Table 1).

### Characteristics of children with algorithm- defined ARDS

The demographic and hospital characteristics of children with algorithm-defined ARDS, based on weighted national estimates, are presented in Table 2. The median age of children with algorithm-defined ARDS was 3 years (IQR 0–16) in 2016, and 2 years (IQR 0–15) in both 2019 and 2022. Males constituted approximately 59% of algorithm-defined ARDS hospitalizations across all years. Using the most recent data from 2022, race and/or ethnicity were missing in 2641 (9.2%) children. Among the non-missing children, the race and/or ethnicity distribution was 43.9% White, 22.1% Black, 22.1% Hispanic, and 11.8% Other (Table 2). The largest proportion of children with algorithm-defined ARDS belonged to households in the lowest median income quartile (33.2%). The Southern US region accounted for the largest proportion of algorithm-defined ARDS hospitalizations (39.9%). The vast majority of algorithm-defined ARDS care occurred in urban teaching hospitals (96.3%). The median length of stay was 11 (2016, 2019)–12 (2022) days (Supplemental Figure S3a), and median total charges showed an increasing trend, reaching $221,508 (IQR $98,710–$543,210) in 2022 (p < 0.001) (Supplemental Figure S3b).

**Table 2:** Weighted inpatient pediatric algorithm- defined ARDS hospitalization characteristics by year.

Hospitalization characteristic	2016	2019	2022
**Age (years)** [median (IQR^a^)]	3 (0, 16)	2 (0, 15)	2 (0, 15)
**Length of stay** [median (IQR^a^)]	11 (5, 25)	11 (5, 25)	12 (5, 26)
**Total charges ($)** (Inflation adjusted to 2022 dollars) [median (IQR^a^)]	$186,477.80 (78,552.52–450,989.10)	$198,870.50 (87,753.45–489,799.80)	$221,507.50 (98,709,895–543,210)
**Biologic sex**			
Female	41.0% (16,332)	41.4% (16,332)	41.2% (16,166)
Male	59.0% (23,237)	58.6% (23,164)	58.8% (23,105)
**Hospital region**			
Northeast	13.8% (5437)	13.2% (5206)	13.9% (5456)
Midwest	23.1% (9094)	24.9% (9824)	24.5% (9616)
South	41.3% (16,278)	40.8% (16,124)	39.9% (15,687)
West	21.8% (8576)	21.1% (8355)	21.7% (8538)
**Race/Ethnicity**			
White	47.6% (16,623)	45.3% (16,526)	43.9% (15,675)
Black	20.9% (7304)	21.9% (7963)	22.1% (7874)
Hispanic	21.1% (7379)	22.1% (7816)	22.1% (7895)
Other	10.3% (3601)	11.4% (4140)	11.8% (4224)
**Hospital location**			
Rural	1.1% (439)	1.0% (398)	1.1% (437)
Urban (Non-Teaching)	7.0% (2760)	3.3% (1292)	2.6% (1034)
Urban (Teaching)	91.9% (36,184)	95.7% (37,819)	96.3% (37,825)
**Hospital bed size**			
Small	9.4% (3699)	11.4% (4486)	11.5% (4538)
Medium	22.8% (8977)	20.0% (7893)	19.9% (7806)
Large	67.8% (26,708)	68.7% (27,131)	68.6% (26,953)
**Median household income quartile**			
0–25th	34.2% (13,228)	34.5% (13,416)	33.2% (12,898)
26th–50th	25.0% (9675)	25.4% (9860)	26.4% (10,269)
51st–75th	23.2% (8965)	23.0% (8937)	22.8% (8856)
76th–100th	17.6% (6820)	17.1% (6653)	17.6% (6825)

a IQR = Interquartile Range.

### Risk factors for algorithm- defined ARDS

In non-birth hospitalizations, the presence of at least one CCC at time of discharge was common and increased in each successive KID iteration, affecting 73.5% of algorithm-defined ARDS patients in 2016 and increasing to 75.6% by 2022. Mortality in this subgroup was high (Supplemental Table S2). The prevalence of immunodeficiency amongst algorithm-defined ARDS patients increased from 2.1% (95% CI 1.9–2.2) in 2016 to 5.1% (4.8–5.3) in 2022 with mortality in these patients also increasing from 15.6% (95% CI 12.9–18.8%) in 2016 to 20.4% (95% CI 18.4–22.5%) in 2022.

### Geographical and racial and/or ethnic disparities in algorithm- defined ARDS mortality

**Fig. 1:**
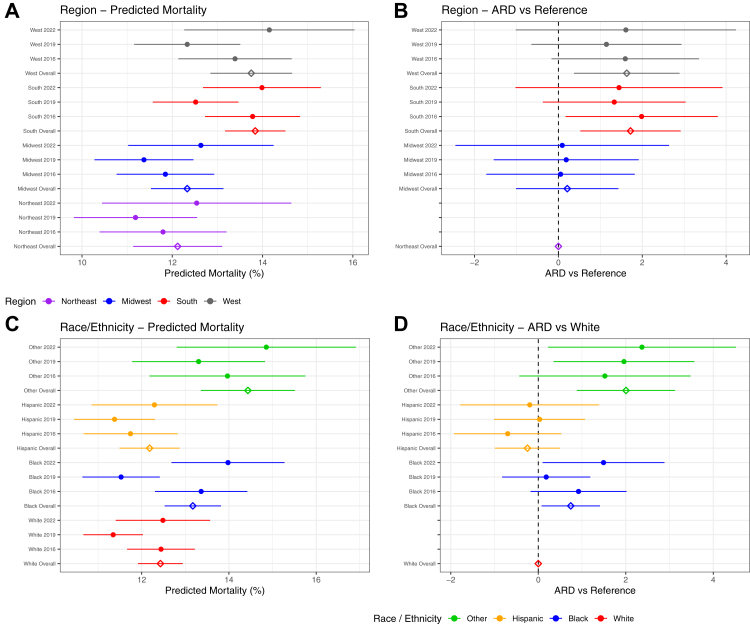
(A–D): Adjusted Predicted Mortality and Risk Differences for a patient with Algorithm- Defined ARDS by United States Region and Race and/or Ethnicity. Fig. 1A demonstrates confounder adjusted predicted mortality by US Region for each region yearly by KID release (2016, 2019, 2022) and overall (all three KID releases combined). Fig. 1B demonstrates the absolute risk difference for mortality compared to the reference (Northeast). Color legend for Fig. 1A–B is in Fig. 1A. Fig. 1C demonstrates confounder adjusted predicted mortality by Race and/or Ethnicity yearly by KID release (2016, 2019, 2022) and overall (all three KID releases combined). Fig. 1D demonstrates the absolute risk difference for mortality compared to the reference (White). Color legend for Fig. 1C–D is in Fig. 1C.

Northeastern children had a confounder adjusted probability of mortality from algorithm-defined ARDS of 12.1% (95% CI 11.1–13.1) when the 3 KID iterations were combined. Regional differences in mortality existed with both admission in the South (ARD 1.72%, 0.52–2.92) or West (ARD 1.63%, 0.37–2.89) regions being associated with increased mortality (Fig. 1A–B) (Supplemental Table S3). When evaluating each year individually, admission in the South in 2016 was associated with increased mortality. Admission in the Midwest was not associated with increased mortality, compared to the Northeast in either yearly or combined analyses.

Confounder adjusted predicted mortality for White children with algorithm-defined ARDS when the 3 iterations of KID were combined was 12.43% (95% CI 11.92–12.94). Disparities in mortality risk according to race and/or ethnicity were present for Black patients with algorithm-defined ARDS (ARD 0.74%, 95% CI 0.08–1.41) but only significantly higher in 2022 for individual-year analyses (Fig. 1C–D) (Supplemental Table S4). “Other” race and/or ethnicity had higher odds of mortality in 2019, 2022, and when all years were combined. Hispanic mortality did not differ in either yearly or combined analyses. Imputed results for missing race and/or ethnicity variables were similar to results of the complete case analysis (Supplemental Table S5).

**Fig. 2:**
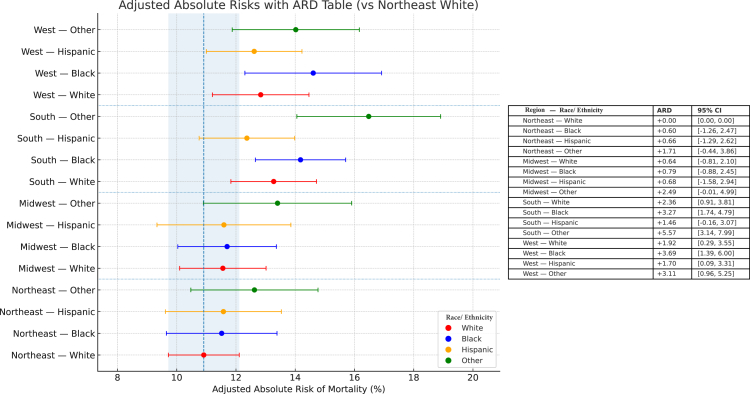
Combined Adjusted Predicted Mortality and Risk Differences for the Joint Exposure of Race and/or Ethnicity and Hospitalization Region. The three iterations of KID (2016, 2019, 2022) were combined to determine predicted mortality of Algorithm- Defined ARDS adjusted for Zip Income Quartile, APRDRG Severity, Hospital Type, and Presence of Complex Chronic Condition. Risk Differences for the joint exposure of Race and/or Ethnicity and hospitalization region are shown in the table on the right side of the figure. For clarity, regions are grouped together and Race and/or Ethnicity is indicated by the color legend in the bottom right corner of the figure.

**Fig. 3:**
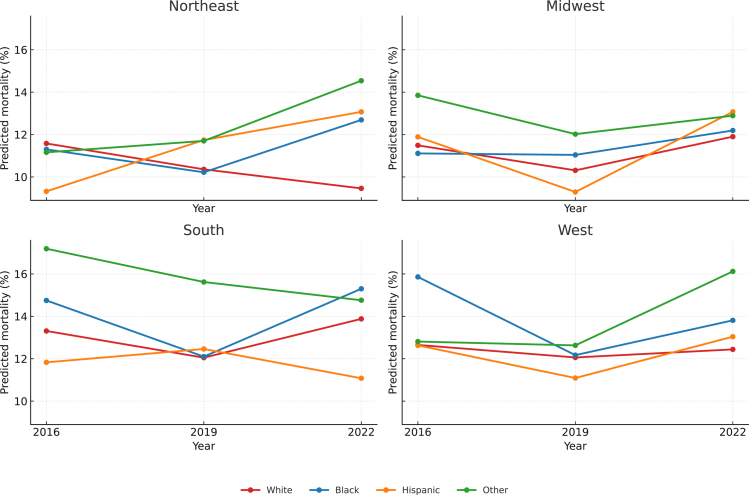
Adjusted Predicted Mortality Trend for the Joint Exposure of Race and/or Ethnicity and Hospitalization Region by Year (2016, 2019, 2022).

The confounder adjusted predicted mortality of the joint model reference group, Northeastern White children, was 10.9% (95% CI 9.72–12.1). There were associated disparities in mortality for Black children in the South (Predicted Mortality 14.2%, ARD 3.27, 1.74–4.79) and West (Predicted Mortality 14.6%, ARD 3.69, 1.39–6.00); Hispanic children in the West (Predicted Mortality 12.6%, ARD 1.70, 0.09–3.31); and children in the “Other” race and/or ethnicity group in the South (Predicted Mortality 16.5%, ARD 5.57, 3.14–7.99), and West (Predicted Mortality 14.0%, ARD 3.11, 0.96–5.25) (Fig. 2) (Supplemental Table S6). Again, imputed results for missing race and/or ethnicity variables were similar to results of the complete case analysis (Supplemental Table S7). Additionally, being admitted for algorithm-defined ARDS as a White child in the South (Predicted Mortality 13.3%, ARD 2.36, 0.91–3.81) and West (Predicted Mortality 12.8%, ARD 1.92, 0.29–3.55) were associated with higher risk of mortality. When restricting to each KID iteration, Southern Black children had increased risk of mortality in 2016 (ARD 3.17, 95% CI 0.61–5.73) that was attenuated in 2019 (ARD 1.74%, 95% CI −0.43 to 3.92), before becoming significant again in 2022 (ARD 5.84%, 95% CI 2.86–8.82) (Fig. 3) (Supplemental Table S8).

The trend in mortality from 2016 to 2019 and separately, 2019 to 2022, was evaluated using the interaction of race/ethnicity or US region with year. The overall predicted mortality between 2016 and 2019 decreased by 0.57% (95% CI −1.36 to 0.22, p = 0.16), a non-significant decline. By race/ethnicity group, the estimated changes were White −0.67% (95% CI −1.7 to 0.35; p = 0.20), Black −1.27% (95% CI −2.64 to 0.10; p = 0.069), Hispanic 0.15% (95% CI −1.27 to 1.57; p = 0.84), and Other −0.14% (95% CI −2.46 to 2.18; p = 0.91). There was no association between race/ethnicity and predicted mortality change as evaluated by the interaction term between race/ethnicity and time being non-significant (χ^2^ = 2.32, p = 0.51). From 2019 to 2022, adjusted predicted mortality increased by 9.69% (95% CI 0.082–0.111; p < 0.001). Increases were similar across races—White 9.18% (95% CI 7.5–10.8), Black 10.9% (95% CI 9.0–12.8), Hispanic 8.91% (95% CI 6.9–10.9), Other 10.8% (95% CI 8.0–13.5). As with 2016–2019, the interaction of race/ethnicity and time was not significant (χ^2^ = 3.14, p = 0.37), indicating no detectable change in racial disparities over this interval.

Within regions from 2016 to 2019, changes in predicted mortality were small and non-significant (Supplemental Table S9). Disparities between regions did not change over this interval (joint test of region × time: χ^2^ (3) = 0.44, p = 0.93). Predicted mortality increased markedly across all regions from 2019 to 2022. By region: Northeast 9.4% (95% CI 6.8–12.0), Midwest 9.2% (95% CI 7.0–11.5), South 10.2% (95% CI 8.2–12.1), and West 10.4% (95% CI 8.0–12.8) (all p < 0.001). Despite these parallel increases, regional disparities remained stable (region × time χ^2^ (3) = 0.09, p = 0.99).

## Discussion

Algorithm-defined ARDS, a ICD-10 coding surrogate for PARDS, occurred in about 1.8% of non-birth pediatric hospitalizations, in a US nationally representative dataset, HCUP KID, during each of the triennial releases from 2016 to 2022, affecting over 42,000 children yearly and was highly fatal with over 12% of those with algorithm-defined ARDS dying during their acute hospitalization in 2022. Prevalence and mortality appeared to be worsening between 2016 and 2022. We found persistent disparities in both US region and race and/or ethnicity mortality outcomes for children with algorithm-defined ARDS between 2016 and 2022. Utilizing joint modeling to explore the intersection of regional and race and/or ethnicity disparities further highlighted disparate outcomes for children of all racial backgrounds in the South and West. After adjustment for socioeconomic factors, illness severity, hospital type, and complex chronic conditions, Black children in the Southern and Western US and children of “Other” race/ethnicity in multiple regions, continued to experience significantly higher risk of algorithm-defined ARDS mortality compared to White children in the Northeast. Furthermore, disparities exist between White children in the South and West compared to Northeastern White children. In some regions, disparities according to race and/or ethnicity appeared to worsen over the study period.

In contrast to the multicenter, multinational PARDIE study,^2^ which derived incidence and outcome data from a convenience sample of participating PICUs across six continents, the present analysis was restricted to U.S. hospitals and applies discharge-level survey weights to KID to yield prevalence estimates that are nationally representative of U.S. pediatric hospitalizations. This methodological distinction not only mitigates geographic heterogeneity but also allows our findings to inform domestic health-system planning and resource allocation more directly. The lower mortality observed in our cohort may signal ongoing improvements in the recognition and management of PARDS within U.S. centers^1^ or inherent differences between algorithm-defined ARDS and true PARDS as defined by PALICC. Consequently, for algorithm defined ARDS, the median length-of-stay (LOS) and total hospitalization charges increased with each triennial release. Increasing charges may represent simply increasing LOS, which may be due to or as a result of increasing proportions of algorithm-defined ARDS patients having comorbidities, CCCs, and immunodeficiency diagnoses. Additionally, the increasing proportion of algorithm-defined ARDS cared for at urban teaching hospitals that increasingly use costly rescue therapies such as extracorporeal membrane oxygenation may contribute to increasing LOS and costs.

Publications highlighting concerns that disparities in care and outcomes for sepsis originated over 20 years ago^11^ and have been common in both adult and pediatric literature for over ten years.^12^^,^^13^ Our group has previously published on pediatric sepsis^6^ and community-acquired pneumonia^7^ revealing disparities connected to racial and geographic factors. Previously hypothesized mechanisms for these disparities include systemic inequalities such as differences in healthcare infrastructure, including access to tertiary and quaternary care, referral systems, and both implicit and explicit biases.^6^^,^^7^^,^^14^ Over the period of study, there was a further concentration of algorithm-defined ARDS care at large, urban, teaching hospitals. Regional disparities in rapid access to these hospitals may contribute to ongoing disparities. Additionally, race and ethnicity differences in the timing of referral and transfer should also be examined. Additionally, factors external to the healthcare system, including the patient's current neighborhood^12^^,^^15^^,^^16^ may be important drivers of health outcomes. Prior to this work, these disparities had not been evaluated in a population limited to children with ARDS. Additionally, the exponential growth in recognition of these disparities^10^^,^^17^ prior to and throughout the duration of years evaluated, offered the opportunity to evaluate if this increasing recognition led to any early evidence of mitigation of these disparities. Unfortunately, we find that both race and/or ethnicity and geographic disparities exist in children with algorithm-defined ARDS, and they do not appear to be improving between 2016 and 2022 and have worsened between 2019 and 2022.

The time lag between recognition of a problem and implementation of effective action,^18^^,^^19^ is starkly evident in these findings. Our data demonstrate a failure to translate increased awareness of disparate outcomes amongst critically ill children into tangible improvements in mortality for the most vulnerable. The consistency of these disparities across related conditions and over an extended period suggests systemic failure to ensure equitable outcomes. It is time that we move the narrative beyond simply acknowledging that disparities exist to confronting the reality that both public policy and the healthcare system are not adequately exploring and implementing interventions to mitigate disparities.

The use of joint modeling to further our mechanistic understanding of these disparities highlights some new, key findings. We benchmarked absolute risk differences against Northeastern White children because this subgroup had the lowest observed PARDS mortality highlighting the excess risk that might be mitigated if outcomes approached those already achieved in this subgroup. This choice should not be interpreted as designating Northeastern White children as a normative or biologically “ideal” group; rather, their outcomes serve as a benchmark that may be achievable under more favorable structural and healthcare circumstances. Algorithm-defined ARDS disparities exist across all examined race and/or ethnicity groups in the West and South. Distance to specialized care and referral networks may play a crucial role. PARDS often requires highly specialized care available in tertiary or quaternary PICUs, including advanced mechanical ventilation strategies or extracorporeal membrane oxygenation (ECMO). Children residing in geographically isolated areas or in regions with less-developed pediatric critical care infrastructure may face delays in accessing such care, potentially leading to worse outcomes.^20^^,^^21^ Between 2008 and 2022, nearly 500 pediatric units closed (representing nearly 30% of pediatric units), with rural and micropolitan units most affected.^22^ Continued regionalization of pediatric critical care services could have driven these disparities from 2016 to 2022. These access barriers often intersect with race and socioeconomic status, as minority and low-income populations may be concentrated in areas with fewer specialized resources or face greater hurdles in navigating complex referral systems. The exaggeration of mortality differences among certain groups within the South and West support this hypothesized mechanism.

Bias and racism, both structural and interpersonal, are recognized as fundamental causes of health disparities.^23^^,^^24^ Within the healthcare system, implicit biases among providers can influence clinical decision-making,^25^ transfer patterns,^26^ communication with families,^27^ and the intensity of care offered.^28^ The persistence of disparities in algorithm-defined ARDS mortality after adjusting for illness severity and available, albeit imperfect surrogates for socioeconomic status, suggests that as with sepsis and pneumonia, we must acknowledge that racism may play a role in altering outcomes in children with PARDS.

This study has several key strengths and limitations. It utilizes a large, nationally representative database, KID, allowing for robust and generalizable estimates of ARDS prevalence, mortality, and associated disparities across the US in children. Our ICD-10 based algorithm for algorithm-defined ARDS identification^4^ limits selection bias that may have occurred in previous voluntary participation incidence studies, but is subject to potential ascertainment bias, as coding practices may vary systematically between academic and non-academic institutions. Administrative data, however, lack the granular clinical details that are integral to the clinical PALICC definition of PARDS,^29^ and we are identifying a population limited to those receiving invasive mechanical ventilation for ≥24 h. Our prevalence and mortality estimates do not include patients presenting in extremis who died within 24 h which may result in underestimation of prevalence and bias estimates of disparities However, our algorithm-defined ARDS estimates reflect a population most amenable to future interventional work to improve outcomes once interacting with the healthcare system. Future work, that may be integral to reducing disparities, focused on PARDS patients dying within 24 h of admission, will require separate identification mechanisms for PARDS. Patient-reported race and ethnicity data in administrative datasets can be imperfect and may not fully capture the complexities of self-identification or multiracial identities. Both the Hispanic and “Other” race/ethnicity categories are heterogeneous, which may mask differing risks among the constituent subgroups. The “Other” race and/or ethnicity category comprised Native American, Asian and Pacific Islander, and “Other” not further specified. This heterogeneous combination of small subgroups means the observed higher mortality should be interpreted at the group level and may mask important disparities within individual subgroups. Race and/or ethnicity was incompletely reported in KID. The proportion of missingness was similar across hospital bed size and region strata but some hospitals had no race and/or ethnicity recorded for any admission, suggesting site-level reporting practices exist. If hospitals that disproportionately serve racial and ethnic minoritized populations are more likely to have incomplete documentation, our complete-case analyses could have underestimated the burden of algorithm-defined ARDS. Multiple imputation analyses produced effect estimates that were highly consistent with the complete-case results, however, multiple imputation cannot fully address data that are truly missing not at random. ZIP codes are large administrative geographic units that may not adequately reflect the median income of individuals residing within that area. In addition, the use of ZIP code median income quartile is an area-level proxy for individual socioeconomic status and is subject to ecological fallacy. Although males comprised a larger proportion of ARDS admissions, we did not adjust for sex because analyses were a priori focused on race and/or ethnicity and US region disparities; therefore, we cannot exclude residual confounding or effect modification by sex and future studies should evaluate sex-specific differences. The analysis spans multiple recent years (2016, 2019, 2022), enabling an assessment of temporal trends. The use of a joint exposure model for race and/or ethnicity and US region allows for a nuanced examination of how these factors interact to influence mortality. Furthermore, the adjustment for key confounders, including a proxy for socioeconomic status (ZIP code median income quartile), illness severity (APRDRG score), hospital type, and the presence of CCCs, strengthens the interpretation of the disparity findings.

Prospective studies collecting granular clinical, process-of-care, geographic, and patient-experience utilizing qualitative or mixed-methods design data are needed to pinpoint critical points of failure in the care continuum for affected minoritized groups, especially for patients dying within 24 h. Community-participatory based intervention and health equity implementation framework designed^30^ studies that specifically target the identified drivers of disparity are paramount. Further investigation into the impact of specific hospital characteristics (e.g., PICU volume, staffing ratios, availability of advanced technologies like ECMO, and culturally competent family support services) on PARDS disparities is also warranted.

### Conclusion

Algorithm-defined ARDS continues to be a significant cause of critical illness and mortality among children hospitalized in the United States. Substantial geographical and racial and/or ethnic disparities in algorithm-defined ARDS mortality exist and showed no improvement between 2016 and 2022. These findings underscore a critical failure to translate awareness of health disparities into effective action for vulnerable pediatric populations over the last 10 years. Urgent, multifaceted, and targeted efforts at clinical, institutional, and policy level interventions are imperative to understand and dismantle the systemic drivers of these disparities.

## Contributors

Garrett Keim: Conceptualization, data curation, formal analysis, methodology, administration, visualization, writing- original draft.

Paula Magee: methodology, writing-review & editing.

Cody Gathers: methodology, writing-review & editing.

Anireddy R. Reddy: methodology, writing-review & editing.

Charlotte Z. Woods-Hill: methodology, writing-review & editing.

Nadir Yehya: Conceptualization, data curation, formal analysis, methodology, supervision, validation, writing-review & editing.

All authors have read and approved the final version of the manuscript. GK and NY accessed and verified the underlying data and analyses. GK had access to raw data and was the final submitter of completed manuscript.

## Data sharing statement

All data used for this study is available from Healthcare Cost and Utilization Project (HCUP) Kids’ Inpatient Database (KID) after completion of a data use agreement. Available online at https://hcup-us.ahrq.gov/tech_assist/centdist.jsp.

## Declaration of generative AI and AI-assisted technologies in the writing process

During the preparation of this work the author(s) used ChatGPT by OpenAI to assist with grammatical and formatting of the manuscript and statistical coding assistance. After using this tool/service, the author(s) reviewed and edited the content as needed and take(s) full responsibility for the content of the publication.

## Declaration of interests

PM declares an NICHD Loan Repayment Award (LRP) and APA Rapid Grant. NY declares grants from NHLBI, NICHD, and NIGMS. GK declares an NHLBI LRP award in addition to the NHLBI K23 that supported this work.
