# Supplementary material for: Geographical and racial and/or ethnic disparities in pediatric ARDS mortality in the USA, 2016–2022: a triennial national database retrospective cohort analysis
Source: Lancet Reg Health Am. 2025 Dec 30;54:101355. doi: 10.1016/j.lana.2025.101355 (PMC12803950; doi:10.1016/j.lana.2025.101355)
Supplement: Supplementary Figures and Tables [file mmc1.docx]

Supplemental Table 1: ICD-10 Codes and Methodologic Algorithm used to Identify Invasively Mechanically Ventilated Pediatric Acute Respiratory Distress Syndrome

| Diagnosis | ICD-10 Codes |
| --- | --- |
| Acute Respiratory Distress Syndrome | J80, J81 |
| Asthma | J45* |
| Pneumonia | J09*, J10*, J11*, J12*, J13*, J14*, J15*, J16*, J17*, J18* |
| Bronchiolitis | J20*, J21*, J22*, J40 |
| Pneumonitis | J68*, J69* |
| Other respiratory failure | J96*, R06.03 |
| Mechanical Ventilation (Diagnosis) | Z99.11 |
| Sepsis/ Shock | A41*, R57*, R65.2* |
| Seizure and Status Epilepticus | G40.901, R56.9*, G40.001, G40.101, G40.111 |
| Procedure | ICD-10 Code |
| Invasive Mechanical Ventilation ≥24 Hours | 5A19045, 5A19055 |
| Algorithm | Code Composition |
| Invasively Mechanically Ventilated Pediatric Acute Respiratory Distress Syndrome | (5A19045 or 5A19055) +  (Any Diagnosis Code for Acute Respiratory Distress Syndrome, Asthma, Pneumonia, Bronchiolitis, Pneumonitis, Other respiratory failure, Sepsis/ Shock) +  (NONE of G40.901 or R56.9*or G40.001 or G40.101 or G40.111) |

Supplemental Table 2: ARDS Prevalence and Mortality by ARDS Risk Factors

| Patient Demographics | 2016 % Prevalence (95% CI) [N] | 2019 % Prevalence (95% CI) [N] | 2022 % Prevalence (95% CI) [N] | 2016 % Mortality (95% CI) [N] | 2019 % Mortality (95% CI) [N] | 2022 % Mortality (95% CI) [N] |
| --- | --- | --- | --- | --- | --- | --- |
| **One or more Complex Chronic Condition at Discharge** | 73.5% (73.0 – 74.0) [28,956] | 74.9% (74.4 – 75.4) [29,584] | 75.6% (75.1 – 76.1) [29,704] | 15.3% (14.9 – 15.8) [4,440] | 14.3% (13.9 – 14.8) [4,241] | 15.6% (15.1 – 16.1) [4,637] |
| **Technology Dependent Complex Chronic Condition at Discharge** | 35.9% (35.3 – 36.4) [14,126] | 38.3% (37.8 – 38.9) [15,138] | 37.3% (36.8 – 37.9) [14,670] | 9.58% (9.02-10.17) [1,353] | 8.70% (8.20-9.24) [1,317] | 9.44% (8.90-10.01) [1,385] |
| **History of Prematurity or Neonatal Complex Chronic Condition** | 11.8% (11.4-12.2) [4,643] | 14.2% (13.8 – 14.6) [5,622] | 15.1% (14.7-15.6) [5,946] | 16.83% (15.59-18.15) [781] | 14.32% (13.29-15.42) [804] | 14.71% (13.69-15.80) [874] |
| **History of Immunodeficiency** | 2.1% (1.9 – 2.2) [813] | 2.0% (1.9 – 2.2) [804] | 5.1% (4.8-5.3) [1,985] | 15.62% (12.88-18.82) [127] | 16.30% (13.54-19.49) [131] | 20.37% (18.37-22.52) [403] |
| **New Tracheostomy during ARDS Hospitalization** | 7.4% (7.1 – 7.7) [2,929] | 6.7% (6.4 – 7.0) [2,659] | 6.5% (6.2 – 6.8) [2,539] | 7.4% (6.4 – 8.6) [217] | 6.5% (5.5 – 7.7) [172] | 7.9% (6.8 – 9.3) [201] |

CI= Confidence Interval, N= weighted number of subjects

Supplemental Table 3: Unadjusted and Adjusted Predicted Probability of Mortality and Absolute Risk Differences by US Region

| Region | Year | Predicted Mortality | LL 95% CI | UL 95% CI | ARD vs base | LL 95% CI | UL 95% CI |
| --- | --- | --- | --- | --- | --- | --- | --- |
|  | **Unadjusted Analyses** | | | | | | |
| Northeast | 2016 | 10.81% | 9.57% | 12.05% |  |  |  |
| Midwest | 2016 | 10.85% | 9.78% | 11.91% | 0.03% | -1.63% | 1.70% |
| South | 2016 | 12.55% | 11.47% | 13.63% | 1.74% | 0.05% | 3.43% |
| West | 2016 | 11.92% | 10.91% | 12.93% | 1.11% | -0.49% | 2.71% |
| Northeast | 2019 | 10.60% | 9.25% | 11.94% |  |  |  |
| Midwest | 2019 | 10.84% | 9.80% | 11.88% | 0.24% | -1.46% | 1.94% |
| South | 2019 | 11.78% | 10.77% | 12.80% | 1.19% | -0.49% | 2.87% |
| West | 2019 | 11.58% | 10.42% | 12.74% | 0.99% | -0.79% | 2.76% |
| Northeast | 2022 | 12.20% | 10.72% | 13.68% |  |  |  |
| Midwest | 2022 | 11.80% | 10.62% | 12.99% | -0.40% | -2.34% | 1.54% |
| South | 2022 | 13.32% | 12.27% | 14.37% | 1.12% | -0.76% | 3.00% |
| West | 2022 | 14.08% | 12.73% | 15.44% | 1.88% | -0.06% | 3.83% |
|  | **Confounder Adjusted Analyses** | | | | | | |
| Northeast | 2016 | 11.79% | 10.39% | 13.20% |  |  |  |
| Midwest | 2016 | 11.85% | 10.77% | 12.93% | 0.05% | -1.72% | 1.82% |
| South | 2016 | 13.78% | 12.73% | 14.83% | 1.98% | 0.17% | 3.80% |
| West | 2016 | 13.39% | 12.13% | 14.65% | 1.60% | -0.16% | 3.35% |
| Northeast | 2019 | 11.19% | 9.82% | 12.55% |  |  |  |
| Midwest | 2019 | 11.37% | 10.28% | 12.47% | 0.19% | -1.54% | 1.92% |
| South | 2019 | 12.52% | 11.57% | 13.47% | 1.33% | -0.37% | 3.04% |
| West | 2019 | 12.33% | 11.16% | 13.51% | 1.15% | -0.64% | 2.94% |
| Northeast | 2022 | 12.54% | 10.44% | 14.64% |  |  |  |
| Midwest | 2022 | 12.63% | 11.02% | 14.24% | 0.09% | -2.45% | 2.64% |
| South | 2022 | 13.99% | 12.68% | 15.29% | 1.45% | -1.02% | 3.91% |
| West | 2022 | 14.15% | 12.26% | 16.04% | 1.61% | -1.02% | 4.23% |

LL=Lower Limit; UL= Upper Limit; ARD= Absolute Risk Difference; CI= Confidence Interval

BASE group for all absolute risk differences was the Northeast region of the corresponding year

Supplemental Table 4: Unadjusted and Adjusted Predicted Probability of Mortality and Absolute Risk Differences by Race and/or Ethnicity

| Race/ Ethnicity | Year | Predicted Mortality | LL 95% CI | UL 95% CI | ARD vs base | LL 95% CI | UL 95% CI |
| --- | --- | --- | --- | --- | --- | --- | --- |
|  | **Unadjusted Analyses** | | | | | | |
| White | 2016 | 11.33% | 10.63% | 12.03% |  |  |  |
| Black | 2016 | 12.22% | 11.22% | 13.21% | 0.88% | -0.16% | 1.92% |
| Hispanic | 2016 | 10.90% | 9.87% | 11.93% | -0.43% | -1.61% | 0.74% |
| Other | 2016 | 13.60% | 11.89% | 15.30% | 2.26% | 0.40% | 4.12% |
| White | 2019 | 10.72% | 10.04% | 11.39% |  |  |  |
| Black | 2019 | 11.33% | 10.40% | 12.27% | 0.62% | -0.41% | 1.64% |
| Hispanic | 2019 | 10.66% | 9.76% | 11.57% | -0.05% | -1.06% | 0.95% |
| Other | 2019 | 12.80% | 11.37% | 14.24% | 2.09% | 0.58% | 3.60% |
| White | 2022 | 12.20% | 11.44% | 12.96% |  |  |  |
| Black | 2022 | 13.78% | 12.78% | 14.79% | 1.59% | 0.50% | 2.67% |
| Hispanic | 2022 | 12.04% | 10.97% | 13.11% | -0.16% | -1.36% | 1.05% |
| Other | 2022 | 14.45% | 13.05% | 15.86% | 2.25% | 0.77% | 3.73% |
|  | **Confounder Adjusted Analyses** | | | | | | |
| White | 2016 | 12.44% | 11.67% | 13.22% |  |  |  |
| Black | 2016 | 13.36% | 12.30% | 14.42% | 0.92% | -0.18% | 2.02% |
| Hispanic | 2016 | 11.74% | 10.67% | 12.82% | -0.70% | -1.92% | 0.53% |
| Other | 2016 | 13.97% | 12.18% | 15.75% | 1.52% | -0.43% | 3.48% |
| White | 2019 | 11.35% | 10.66% | 12.03% |  |  |  |
| Black | 2019 | 11.53% | 10.64% | 12.42% | 0.18% | -0.83% | 1.19% |
| Hispanic | 2019 | 11.38% | 10.45% | 12.31% | 0.03% | -1.01% | 1.07% |
| Other | 2019 | 13.31% | 11.79% | 14.83% | 1.96% | 0.35% | 3.57% |
| White | 2022 | 12.49% | 11.41% | 13.57% |  |  |  |
| Black | 2022 | 13.98% | 12.68% | 15.27% | 1.49% | 0.10% | 2.88% |
| Hispanic | 2022 | 12.29% | 10.85% | 13.73% | -0.19% | -1.78% | 1.39% |
| Other | 2022 | 14.86% | 12.80% | 16.91% | 2.37% | 0.22% | 4.52% |

LL=Lower Limit; UL= Upper Limit; ARD= Absolute Risk Difference; CI= Confidence Interval

BASE group for all absolute risk differences was subjects identified as White in the corresponding year

Supplemental Table 5: Missing Race and/or Ethnicity Imputed Predicted Mortality and Absolute Risk Differences

| Exposure of Interest | Predicted Mortality | LL 95% CI | UL 95% CI | ARD vs. Base | LL 95% CI | UL 95% CI |
| --- | --- | --- | --- | --- | --- | --- |
| White | 12.67% | 12.16% | 13.18% |  |  |  |
| Black | 13.81% | 13.14% | 14.47% | 0.73% | 0.04% | 1.41% |
| Hispanic | 12.73% | 12.02% | 13.45% | -0.21% | -0.97% | 0.54% |
| Other | 15.28% | 14.19% | 16.37% | 2.16% | 1.06% | 3.27% |

LL=Lower Limit; UL= Upper Limit; ARD= Absolute Risk Difference; CI= Confidence Interval

BASE group for all absolute risk differences was subjects identified as White

Supplemental Table 6: Confounder Adjusted Predicted Mortalities and Absolute Risk Differences by US Region, Race and/or Ethnicity, and the Joint Exposure Model for all Years 2016, 2019, and 2022.

| Exposure | Predicted Mortality | LL 95% CI | UL 95% CI | ARD vs Base | LL 95% CI | UL 95% CI |
| --- | --- | --- | --- | --- | --- | --- |
|  | **US Region** | | | | | |
| Northeast | 12.12% | 11.13% | 13.10% |  |  |  |
| Midwest | 12.33% | 11.53% | 13.13% | 0.21% | -1.01% | 1.43% |
| South | 13.84% | 13.17% | 14.51% | 1.72% | 0.52% | 2.92% |
| West | 13.75% | 12.84% | 14.66% | 1.63% | 0.37% | 2.89% |
|  | **Race and/or Ethnicity** | | | | | |
| White | 12.43% | 11.92% | 12.94% |  |  |  |
| Black | 13.17% | 12.53% | 13.82% | 0.74% | 0.08% | 1.41% |
| Hispanic | 12.18% | 11.49% | 12.87% | -0.25% | -0.99% | 0.49% |
| Other | 14.43% | 13.36% | 15.51% | 2.01% | 0.88% | 3.13% |
|  | **Joint Exposure Model** | | | | | |
| Northeast White | 10.91% | 9.72% | 12.11% |  |  |  |
| Northeast Black | 11.52% | 9.87% | 13.16% | 0.60% | -1.26% | 2.47% |
| Northeast Hispanic | 11.58% | 9.75% | 13.40% | 0.66% | -1.29% | 2.62% |
| Northeast Other | 12.62% | 10.77% | 14.48% | 1.71% | -0.44% | 3.86% |
| Midwest White | 11.56% | 10.67% | 12.44% | 0.64% | -0.81% | 2.10% |
| Midwest Black | 11.70% | 10.53% | 12.87% | 0.79% | -0.88% | 2.45% |
| Midwest Hispanic | 11.59% | 9.65% | 13.53% | 0.68% | -1.58% | 2.94% |
| Midwest Other | 13.40% | 11.19% | 15.62% | 2.49% | -0.01% | 4.99% |
| South White | 13.27% | 12.46% | 14.09% | 2.36% | 0.91% | 3.81% |
| South Black | 14.18% | 13.24% | 15.12% | 3.27% | 1.74% | 4.79% |
| South Hispanic | 12.37% | 11.27% | 13.47% | 1.46% | -0.16% | 3.07% |
| South Other | 16.48% | 14.33% | 18.63% | 5.57% | 3.14% | 7.99% |
| West White | 12.83% | 11.67% | 14.00% | 1.92% | 0.29% | 3.55% |
| West Black | 14.61% | 12.61% | 16.61% | 3.69% | 1.39% | 6.00% |
| West Hispanic | 12.61% | 11.48% | 13.75% | 1.70% | 0.09% | 3.31% |
| West Other | 14.02% | 12.19% | 15.84% | 3.11% | 0.96% | 5.25% |

LL=Lower Limit; UL= Upper Limit; ARD= Absolute Risk Difference; CI= Confidence Interval

BASE for the US Region analysis was Northeast; BASE for the Race and/or Ethnicity analyses was White; BASE for the Joint Exposure Model was subjects identified as White in the Northeast

Supplemental Table 7: Missing Race and/or Ethnicity Imputed Predicted Mortality and Absolute Risk Differences for all Years 2016, 2019, and 2022

| Joint Exposure of Race and Region | Predicted Mortality | LL 95% CI | UL 95% CI | ARD vs NE White | LL 95% CI | UL 95% CI |
| --- | --- | --- | --- | --- | --- | --- |
|  |  |  |  |  |  |  |
| Northeast White | 11.00% | 9.79% | 12.19% |  |  |  |
| Northeast Black | 12.14% | 10.45% | 13.84% | 0.82% | -1.14% | 2.78% |
| Northeast Hispanic | 12.35% | 10.45% | 14.25% | 0.68% | -1.33% | 2.70% |
| Northeast Other | 14.42% | 12.36% | 16.48% | 2.09% | -0.13% | 4.32% |
| Midwest White | 12.08% | 11.18% | 12.99% | 0.62% | -0.90% | 2.14% |
| Midwest Black | 12.71% | 11.45% | 13.97% | 0.80% | -0.92% | 2.53% |
| Midwest Hispanic | 12.32% | 10.37% | 14.27% | 0.66% | -1.59% | 2.91% |
| Midwest Other | 14.40% | 12.11% | 16.69% | 2.65% | 0.11% | 5.19 |
| South White | 13.32% | 12.49% | 14.14% | 1.98% | 0.46% | 3.50% |
| South Black | 14.58% | 13.61% | 15.54% | 2.83% | 1.22% | 4.44% |
| South Hispanic | 12.66% | 11.53% | 13.79% | 1.27% | -0.41% | 2.95% |
| South Other | 16.68% | 14.56% | 18.81% | 5.18% | 2.72% | 7.64% |
| West White | 13.26% | 12.07% | 14.44% | 2.02% | 0.32% | 3.71% |
| West Black | 15.40% | 13.26% | 17.54% | 3.86% | 1.42% | 6.29% |
| West Hispanic | 13.41% | 12.19% | 14.62% | 1.68% | 0.00% | 3.36% |
| West Other | 14.90% | 13.01% | 16.79% | 3.31% | 1.12% | 5.49% |

LL=Lower Limit; UL= Upper Limit; ARD= Absolute Risk Difference; CI= Confidence Interval; NE = Northeast

Supplemental Table 8: Adjusted Predicted Probability of Mortality and Absolute Risk Differences by Race and/or Ethnicity

| Joint Exposure of Race and Region | Year | Predicted Mortality | LL 95% CI | UL 95% CI | ARD vs NE White | LL 95% CI | UL 95% CI |
| --- | --- | --- | --- | --- | --- | --- | --- |
|  | **2016** | | | | | | |
| Northeast White | 2016 | 11.58% | 9.59% | 13.57% |  |  |  |
| Northeast Black | 2016 | 11.30% | 8.77% | 13.83% | -0.28% | -3.32% | 2.76% |
| Northeast Hispanic | 2016 | 9.32% | 6.53% | 12.10% | -2.26% | -5.45% | 0.92% |
| Northeast Other | 2016 | 11.16% | 8.20% | 14.12% | -0.42% | -3.88% | 3.04% |
| Midwest White | 2016 | 11.49% | 10.29% | 12.68% | -0.10% | -2.41% | 2.22% |
| Midwest Black | 2016 | 11.11% | 9.27% | 12.95% | -0.47% | -3.19% | 2.25% |
| Midwest Hispanic | 2016 | 11.89% | 8.60% | 15.17% | 0.30% | -3.53% | 4.14% |
| Midwest Other | 2016 | 13.85% | 10.53% | 17.16% | 2.27% | -1.60% | 6.14% |
| South White | 2016 | 13.31% | 11.97% | 14.65% | 1.73% | -0.69% | 4.15% |
| South Black | 2016 | 14.75% | 13.18% | 16.33% | 3.17% | 0.61% | 5.73% |
| South Hispanic | 2016 | 11.83% | 10.04% | 13.62% | 0.25% | -2.40% | 2.89% |
| South Other | 2016 | 17.19% | 13.20% | 21.19% | 5.61% | 1.24% | 9.99% |
| West White | 2016 | 12.65% | 10.90% | 14.39% | 1.07% | -1.52% | 3.65% |
| West Black | 2016 | 15.86% | 12.63% | 19.08% | 4.27% | 0.51% | 8.04% |
| West Hispanic | 2016 | 12.63% | 10.86% | 14.41% | 1.05% | -1.55% | 3.66% |
| West Other | 2016 | 12.81% | 10.09% | 15.53% | 1.23% | -2.13% | 4.59% |
|  | **2019** | | | | | | |
| Northeast White | 2019 | 10.36% | 8.62% | 12.10% |  |  |  |
| Northeast Black | 2019 | 10.22% | 7.80% | 12.64% | -0.14% | -2.93% | 2.65% |
| Northeast Hispanic | 2019 | 11.74% | 9.04% | 14.44% | 1.38% | -1.45% | 4.21% |
| Northeast Other | 2019 | 11.70% | 9.10% | 14.30% | 1.34% | -1.79% | 4.47% |
| Midwest White | 2019 | 10.31% | 9.12% | 11.50% | -0.05% | -2.15% | 2.04% |
| Midwest Black | 2019 | 11.04% | 9.30% | 12.79% | 0.68% | -1.79% | 3.15% |
| Midwest Hispanic | 2019 | 9.29% | 7.37% | 11.21% | -1.07% | -3.64% | 1.51% |
| Midwest Other | 2019 | 12.02% | 8.58% | 15.45% | 1.66% | -2.21% | 5.52% |
| South White | 2019 | 12.05% | 10.99% | 13.10% | 1.69% | -0.36% | 3.73% |
| South Black | 2019 | 12.10% | 10.84% | 13.36% | 1.74% | -0.43% | 3.92% |
| South Hispanic | 2019 | 12.46% | 10.75% | 14.16% | 2.09% | -0.36% | 4.55% |
| South Other | 2019 | 15.62% | 12.57% | 18.67% | 5.26% | 1.74% | 8.78% |
| West White | 2019 | 12.06% | 10.37% | 13.74% | 1.70% | -0.69% | 4.08% |
| West Black | 2019 | 12.17% | 9.64% | 14.69% | 1.80% | -1.26% | 4.87% |
| West Hispanic | 2019 | 11.09% | 9.67% | 12.50% | 0.72% | -1.52% | 2.97% |
| West Other | 2019 | 12.63% | 10.20% | 15.06% | 2.27% | -0.69% | 5.23% |
|  | **2022** | | | | | | |
| Northeast White | 2022 | 9.46% | 7.06% | 11.85% |  |  |  |
| Northeast Black | 2022 | 12.69% | 9.12% | 16.25% | 3.23% | -0.62% | 7.08% |
| Northeast Hispanic | 2022 | 13.07% | 9.61% | 16.53% | 3.62% | -0.09% | 7.32% |
| Northeast Other | 2022 | 14.54% | 11.19% | 17.88% | 5.08% | 1.12% | 9.05% |
| Midwest White | 2022 | 11.90% | 10.15% | 13.64% | 2.44% | -0.50% | 5.39% |
| Midwest Black | 2022 | 12.19% | 10.04% | 14.35% | 2.74% | -0.50% | 5.97% |
| Midwest Hispanic | 2022 | 13.07% | 8.38% | 17.76% | 3.61% | -1.61% | 8.84% |
| Midwest Other | 2022 | 12.89% | 8.72% | 17.05% | 3.43% | -1.32% | 8.19% |
| South White | 2022 | 13.88% | 12.17% | 15.58% | 4.42% | 1.50% | 7.35% |
| South Black | 2022 | 15.30% | 13.49% | 17.10% | 5.84% | 2.86% | 8.82% |
| South Hispanic | 2022 | 11.08% | 9.15% | 13.02% | 1.63% | -1.43% | 4.69% |
| South Other | 2022 | 14.76% | 11.40% | 18.12% | 5.31% | 1.23% | 9.38% |
| West White | 2022 | 12.44% | 9.91% | 14.97% | 2.98% | -0.41% | 6.38% |
| West Black | 2022 | 13.81% | 9.43% | 18.20% | 4.36% | -0.58% | 9.30% |
| West Hispanic | 2022 | 13.04% | 10.53% | 15.55% | 3.59% | 0.21% | 6.96% |
| West Other | 2022 | 16.12% | 11.85% | 20.39% | 6.67% | 1.87% | 11.46% |

LL=Lower Limit; UL= Upper Limit; ARD= Absolute Risk Difference; CI= Confidence Interval; NE = Northeast

Supplemental Table 9: Absolute Risk Differences in Mortality for US Region and Race and/or Ethnicity comparing 2016 to 2019 and 2019 to 2022.

| Region | Absolute Risk Difference | LL 95% CI | UL 95% CI | p-value |
| --- | --- | --- | --- | --- |
|  | **2019 vs 2016** | | | |
| Northeast | -0.28% | -2.20% | 1.60% | 0.77 |
| Midwest | -0.05% | -1.60% | 1.50% | 0.94 |
| South | -0.76% | -2.30% | 0.80% | 0.327 |
| West | -0.63% | -2.30% | 1.00% | 0.45 |
|  | **2022 vs 2019** | | | |
| Northeast | 9.40% | 6.80% | 12.00% | <0.001 |
| Midwest | 9.20% | 7.00% | 11.00% | <0.001 |
| South | 10.10% | 8.20% | 12.00% | <0.001 |
| West | 10.40% | 8.00% | 13.00% | <0.001 |
|  | **2019 vs 2016** | | | |
| White | -0.67% | -1.70% | 0.35% | 0.2 |
| Black | -1.30% | -2.60% | 0.10% | 0.069 |
| Hispanic | 0.14% | -1.30% | 1.60% | 0.84 |
| Other | -0.14% | -2.50% | 2.20% | 0.91 |
|  | **2022 vs 2019** | | | |
| White | 9.20% | 7.50% | 10.80% | <0.001 |
| Black | 10.90% | 9.00% | 12.80% | <0.001 |
| Hispanic | 8.91% | 6.90% | 10.90% | <0.001 |
| Other | 10.80% | 8.00% | 13.55% | <0.001 |

LL=Lower Limit; UL= Upper Limit; CI= Confidence Interval

Supplemental Figure 1: CONSORT Diagram of yearly unweighted pediatric hospitalizations and PARDS cases, with missing race and/or ethnicity for subjects with and without PARDS.

Supplemental Figure 2: Trend in PARDS Prevalence and Mortality from 2016 through 2022.

Supplemental Figure 3: Algorithm- Defined ARDS Hospitalization Characteristics between 2016 and 2022
